# A Bootstrapped Comparator-Switched Active Rectifying Circuit for Wirelessly Powered Integrated Miniaturized Energy Sensing Systems

**DOI:** 10.3390/s19214714

**Published:** 2019-10-30

**Authors:** Cihun-Siyong Alex Gong, Shiang-Wei Li, Muh-Tian Shiue

**Affiliations:** 1 Department of Electrical Engineering, School of Electrical and Computer Engineering, College of Engineering, Chang Gung University, Taoyuan 33302, Taiwan; sgary830108@gmail.com; 2 Green Technology Research Center, Portable Energy System Group, College of Engineering, Chang Gung University, Taoyuan 33302, Taiwan; 3 Department of Ophthalmology, Chang Gung Memorial Hospital, Linkou Branch, Taoyuan 33304, Taiwan; 4 Department of Electrical Engineering, National Central University, Taoyuan 32001, Taiwan; mtshiue@ee.ncu.edu.tw

**Keywords:** energy harvesting, rectifying circuit, wireless sensing systems, implantable biomedical devices, AC-DC power converters, integrated circuits, RFID tags

## Abstract

Human life expectancy has gradually increased in part through rapid advances in technology, including the development and use of wearable and implantable biomedical electronic devices and sensing monitors. A new architecture is proposed in this paper to replace the traditional diode circuit implementation in wireless power supply systems applied to the above-mentioned devices and monitors. By achieving near-ideal power transistor switching and leveraging the characteristics of conventional diodes to prevent reverse current, the proposed approach greatly improves the performance of the energy harvester in power conversion. The MOS harvester used in the uninterrupted permanent wireless near-field power supply described here for use in biomedical systems was designed and verified using the Taiwan Semiconductor Manufacturing Company (TSMC) standard 180-nm process, achieving performance results of Voltage conversion efficiency (VCE) = 73.55–95.12% and Power conversion efficiency (PCE) = 80.36–90.08% with the output load (0.1–1 kΩ) under 3.3 V ac input with an overall area of 1.189 mm^2^. These results are expected to create an important technical niche for new “green-energy” miniaturized energy sensing systems including cutting edge wirelessly powered biomedical electronics applications.

## 1. Introduction

**Figure 1:**
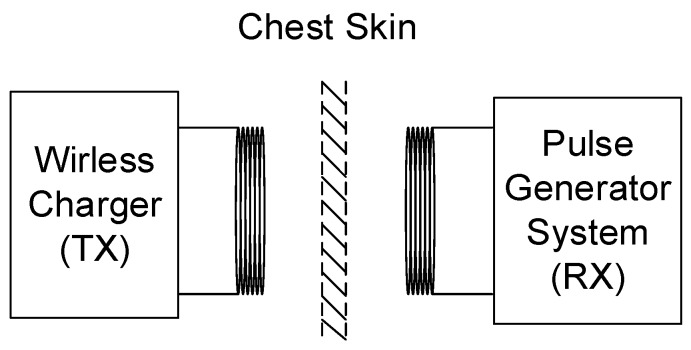
Generic power-combined data transfer system.

In recent years, researchers have made great progress in the development of artificial hearts, ears and eyes [1,2]. However, accelerating the development of practical applications for research into these types of biomedical prostheses depends on improved portability. Most such devices are implanted in the body, raising issues of power supply. For example, the generic implantable power-combined data transfer system shown in Figure 1 uses a wireless charging setup using a power amplifier on the TX end. The coil-resonated transmission generates energy in the form of a sin wave also known as radio frequency wave to the RX end of the secondary side coil, then converts AC to DC through the rectifier, down converts the DC through the LDO, and supplies it to the system for use. The concept is called energy harvesting.

There have been several kinds of ambient energy sources for the energy harvesting applications. In addition to the radio frequency wave, low level mechanical vibrations inherent in the environment can also provide tens to hundreds of microwatts to supply a miniaturized device [3]. In [3], a systematic low-overhead maximum power point (MPP) tracking scheme based on a time-multiplexing mechanism is proposed for micropower energy harvesting system based on mechanical vibrations. It deals with wide frequency range of vibration and load matching resistance variation. It was designed for general piezoelectric conversion, allowing it to be not restricted to cantilever structures. For vibrations, there are also piezoelectric transducers. Unfortunately, the electromechanical coupling factor is low and the generated power is inevitably compromised [4]. The design in [4] deals with the low conversion efficiency of such systems, where it invests harvested energy collected across both the positive half cycle and the battery into the piezoelectric transducer [4]. Since our design along with its applied scenario is based on the radio frequency wave, there will be no battery and vibration sensor involved. The key focus of this type of biomedical system is energy conversion efficiency, which this paper seeks to achieve through the rectifier.

**Figure 2:**
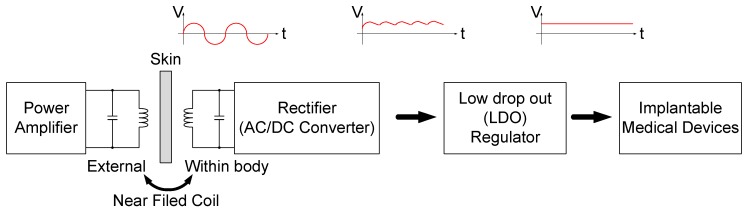
Cardiac pacemaker block diagram.

From the above, wireless charging, such as the architecture shown in Figure 2, must convert AC signals to DC via a rectifier, such as in the rectifier architecture mentioned in [5]. This architecture mainly eliminates the additional threshold voltage (VTH) caused by the substrate effect by dynamically modulating the base voltage, and avoiding the latch-up effect which may cause circuit damage. However, there is no special variability in the main active diode in this architecture, so the resulting conversion performance will not particularly stand out. However, there are many different architectures, such as the mechanism of controlling the lower half of the NMOS cross-coupling pair by using bias voltage and capacitance [6], or by means of bootstrap and a capacitor. VTH can be eliminated to maximize the output voltage VOUT [7,8]. However, the above architecture has no comparator, so the time point of switching to the active diode will be less accurate, resulting in increased reverse current. Therefore, some architectures use the comparator to match some control circuits, and start the pre-charge circuit to help the output terminal reach a voltage value close to that of the DC, and the mode changes with the surrounding digital switching circuit or offset. This improves voltage conversion efficiency, VCE, and power conversion efficiency, PCE, performance [9,10,11,12,13,14,15,16,17,18,19] but, although this method can effectively reduce the reverse current, improvement to VCE and PCE is unsatisfactory, and the circuit takes up a large area.

Some of the above architectures experience performance loss due to excessive reverse currents, and some provide less than ideal conversion performance or circuit areas. Therefore, we propose a new architecture type, with a full n-type power MOSFET, combined with bootstrapped technology and comparator-switched active diodes to address both the area problem and the reverse current problem, while improving VCE and PCE performance, respectively, to 95.12% and 90.08%.

## 2. Bootstrapped Rectifier System Description

**Figure 3:**
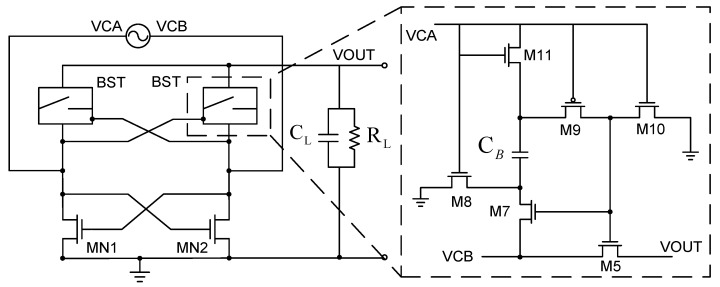
Bootstrap active rectifier circuit [8].

Figure 3 illustrates the bootstrapped rectifier circuit. The bottom left of the figure shows a cross-coupled pair in a double-crossing active rectifier. It operates like a switch without a diode voltage drop, and the upper part of the figure is a bootstrap circuit. Since the NMOS drive outperforms PMOS, it can provide similar current drive capability using less space. However, it can only be charged up to one Vth through NMOS (where Vth is threshold voltage of the NMOS transistor conduction). Therefore, the bootstrap circuit is used to raise the gate voltage of the NMOS above the input maximum voltage by more than one Vth, such that the NMOS can also charge the output capacitor to the highest potential, like the PMOS switch. The right half of the circuit diagram is the bootstrap circuit. In principle, the circuit operates as follows. When VCA is high, VCB is low; M8, M10, and M11 are turned on; M5, M7 and M9 are turned off; and the capacitor CB is charged by VCA. After the high and low states are interchanged, when VCB is in the high state, M8, M10 and M11 are turned off, while M5, M7 and M9 are turned on. The voltage of the M5 gate (VCB + VC_B_) exceeds the maximum input voltage, allowing VOUT to reach VCB with almost no voltage, completely resolving the problem that the bridge rectifier wastes the turn-on voltage. Although this rectifier overcomes the problem of the diode turn-on voltage, it has the same reverse current problem as the double-crossing rectifier. When the input voltage has passed the highest value, the switch is still on, thus it will continue to charge and discharge, causing power loss and reducing conversion efficiency. However, this architecture has a fatal flaw in terms of transistor breakdown. Section 3 proposes an improved breakdown method, mainly by biasing a stable DC value at the output using PMOS as the diode connection configuration. The architecture in Figure 3 shows the M9, M10 and M11 control switches. When VCB is high and VCA is low, the M9, M10 and M11 switches suffer from a VCB+VCB-VCA voltage difference, thus naturally no breakdown occurs. Section 3 provides the detailed architecture and simulation results.

### 2.1. Parasitic Transistor Latch-up Effect

**Figure 4:**
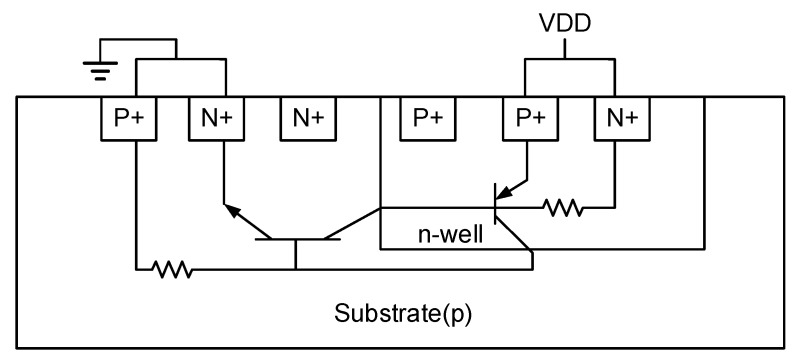
Parasitic transistor [5].

**Figure 5:**
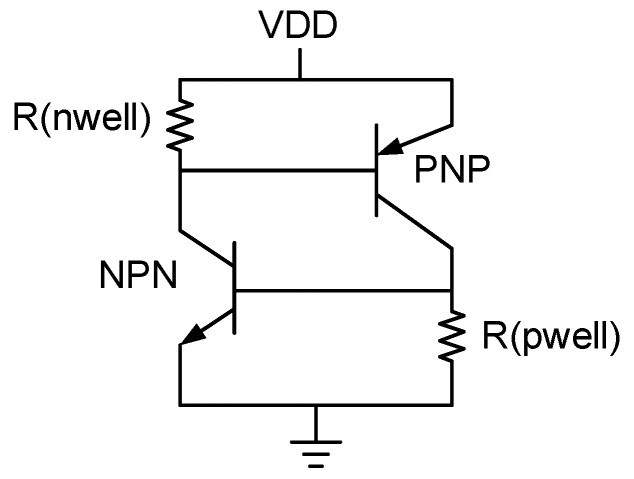
Parasitic CMOS latch equivalent parasitic circuit [5].

The parasitic structure is usually formed by the PNPN stack structure (Figure 4) in the CMOS IC, and the structure of these PNPNs can be further divided into a PNP transistor and a NPN transistor stack. When either the PNP or NPN transistors in the parasitic transistor structure is turned on, the connection also affects the conduction of the other pair of transistors, because they remain associated as long as any transistor forms a forward bias and generates a current, thus the parasitic structure remains until the circuit is turned off (Figure 5). Therefore, the base end of the PNP transistor is too large, or the voltage VDD at the input terminal increases until it is sufficiently biased, and the current conducted at the emitter terminal is directly conducted to the base end of the NPN to activate the NPN transistor. Thus, the current gain is several hundred times the product of the gain of the NPN transistor and the PNP transistor. Under light conditions, this may affect the circuit’s ability to work properly, and under severe conditions, the overall PNP transistor’s current to ground will be excessively large, causing the chip to burn out. Other common causes of the latch-up effect are a positive or negative voltage surge at the input or output, or electrostatic discharge. Therefore, to reduce the impact of the latch-up effect, the material impedance of the P-well is reduced during layout, or the distance of each MOS increased. That is, the distance of P+ is increased to reduce the possibility of parasitic transistor conduction, and also reduce the parasitic effect on the RC. The partial pressure of the VBE terminal of the PNP or the NPN transistor is not excessively increased, and the transistor is biased in the forward direction to be activated.

### 2.2. Dynamic Bulk Switch

**Figure 6:**
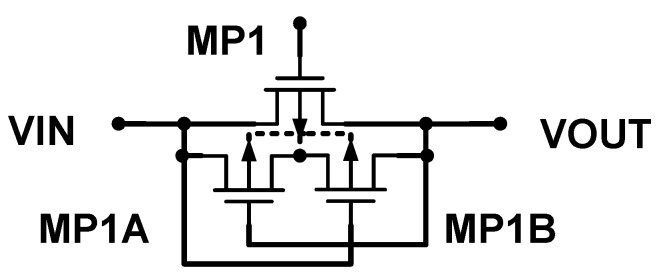
Dynamic bulk modulation [5].

The Dynamic Bulk Switch reduces the CMOS body effect and latch-up effect. The circuit uses a single PMOS power transistor to represent the dynamic body modulation circuit, as shown in Figure 6.

We add a set of small auxiliary PMOS transistors (MP1A and MP1B) to the main power transistor PMOS switch MP1 on the circuit, connecting the source end of the power transistor with the drain end, and then use individual independent N-wells. By cross-controlling the transistor gate, when the source (input) voltage is high, Vin > Vout. At this time, the body terminal of the power transistor PMOS is turned on by MP1A to make its potential equal to the source end of MP1. The power transistor is turned on to charge the RC circuit at the load end. However, when Vin < Vout, the power transistor itself is cut off. But at this time, the RC is charged in the upper half cycle, so the output terminal Vout is still in a high potential state. Thus, the body effect is not eliminated, causing problems including reverse current and leakage current. Therefore, we must eliminate the body effect through the auxiliary transistor MP1B. Thus, MP1B is turned on so that the body terminal of the power transistor MP1 has the same potential as Vout. The VSB can be made consistent to eliminate the body effect. At this time, because the Vout terminal is a relatively high voltage terminal, it is the source. However, another advantage of this circuit is that it can prevent latch-up. If the power transistor is followed by the power transistor NOMS, the two auxiliary transistors (MP1A, MP1B) will not be turned on at the same time, and their source and drain voltages are close to zero, thus preventing the parasitic vertical PNP transistors and also preventing latch-up.

### 2.3. Start Up Rectifier Circuit and Voltage Monitoring Circuit

**Figure 7:**
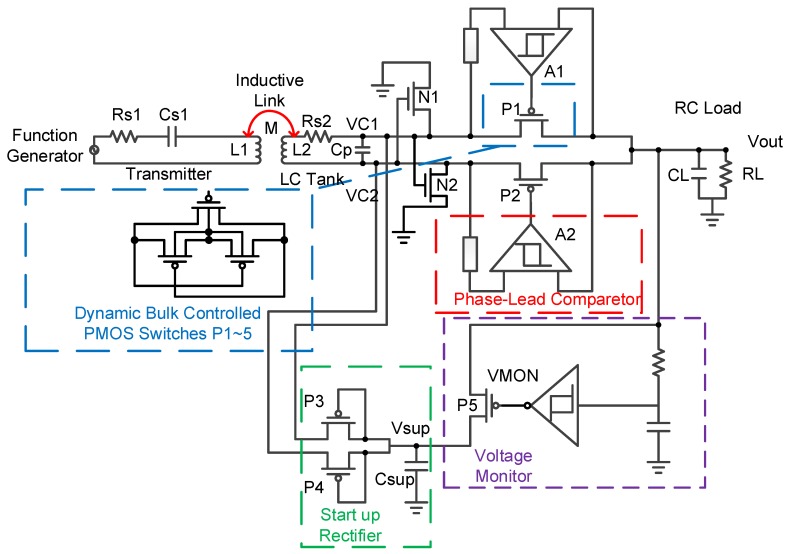
Architecture from [9].

To improve the shortcomings of the voltage collapse of the Figure 3, this paper integrates the functions of the Start Up Rectifier and Voltage Monitor, along with additional circuitry. The two circuits are described in detail as follows. From the rectifier circuit architecture of the reference circuit (Figure 7), we find two circuits that can help address the problem of the bootstrap circuit crash: The Start Up Rectifier and the Voltage Monitor.

**Figure 8:**
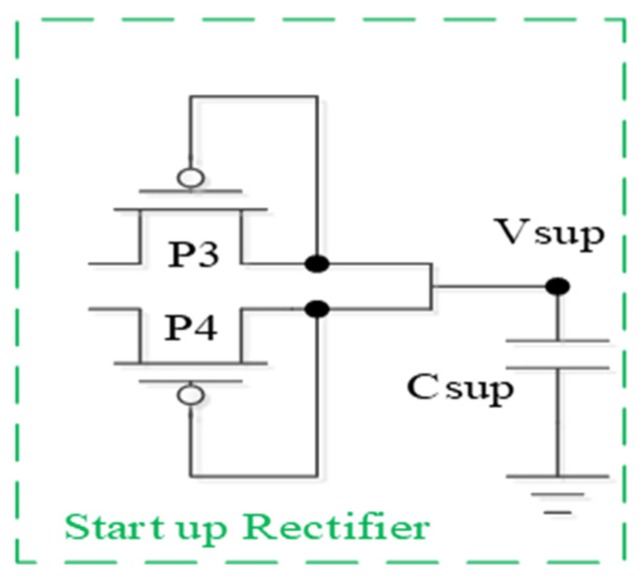
Start Up Rectifier [9].

We start with the Start Up Rectifier circuit (green dotted line in Figure 7 and Figure 8), which is mainly used to pre-charge a capacitor to a certain voltage value. The Vsup can pre-store the voltage before the main circuit is started, at which point the voltage is used to complete the function of the main circuit.

**Figure 9:**
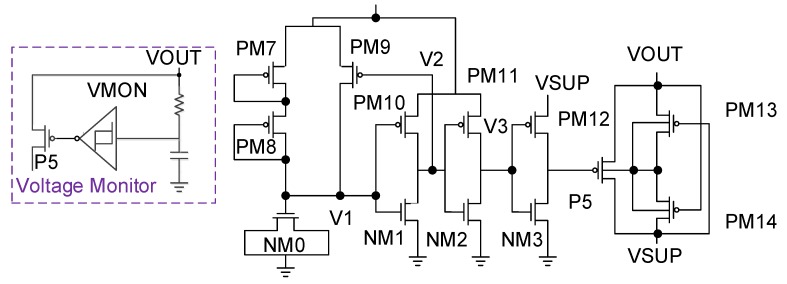
Voltage Monitor [9].

Then, the operation of the Voltage Monitor circuit (the purple line in Figure 7 and Figure 9) is analyzed. This circuit mainly functions to monitor the voltage of Vout to adjust the conduction capability of the P5 PMOS, which is analyzed in Figure 9. From the mid-right side of the figure, PM7 and PM8 are mainly connected in a form of diode to perform partial pressure action and then regulate the voltage with NM0 (MOS capacitor). We then use the PM10 and NM1 inverter to modulate PM9′s PMOS turn-on capability. When V1 is high relative to Vout and VSS, V2 outputs VSS. At this time, the potential of V1 is lower than expected, so PM9 will turn on, causing the node voltage level of V1 to rise. However, when V1 is low relative to Vout and VSS, V2 outputs Vout. At this time, the potential representing V1 is higher than expected, so PM9 will be cut off, thus lowering the node voltage level of V1, achieving characteristics similar to amplifier closed-loop modulation. Finally, PM11 and PM12 and the underlying NMOS are used as a buffer to push the P5 PMOS.

### 2.4. Comparative Rectifier

**Figure 10:**
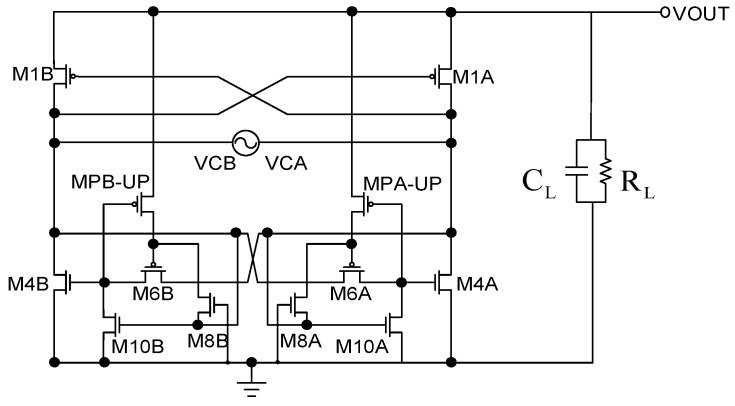
Comparative rectifier [10].

The comparative rectifier (Figure 10) architecture is mainly used to decrease the reverse current. Thus, in the lower part we use a similar comparator concept to immediately cut off M4A or M4B when the VCA or VCB is less than VOUT, so as to reduce the reverse current. However, after the ratio of the reverse current decreases, the overall circuit performance of VCE and PCE will be improved. The circuit architecture is analyzed as follows.

**Figure 11:**
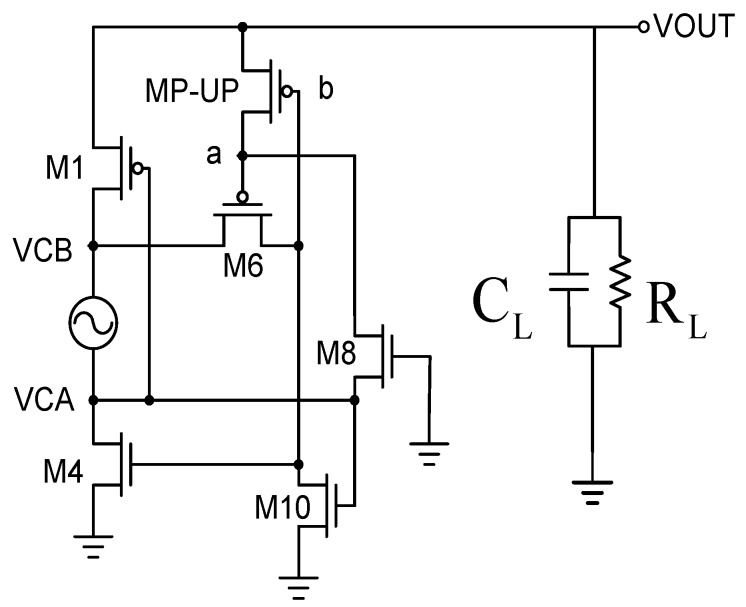
Comparative rectifier equivalent simplified diagram [10].

To facilitate analysis and explain the behavior mode of the circuit, we simplify the circuit as shown in Figure 11. Before analysis, we must first explain that the circuit has two transistors and used a special process. M8, M10, the so-called depleted transistor, is relative to the reinforced transistor. The transistor is manufactured with a channel, allowing it to operate normally even without sufficient voltage or bias. In other words, its threshold voltage value is close to zero, making it completely different from the case where the general reinforced transistor can only be turned on when VGS > VTH.

**Figure 12:**
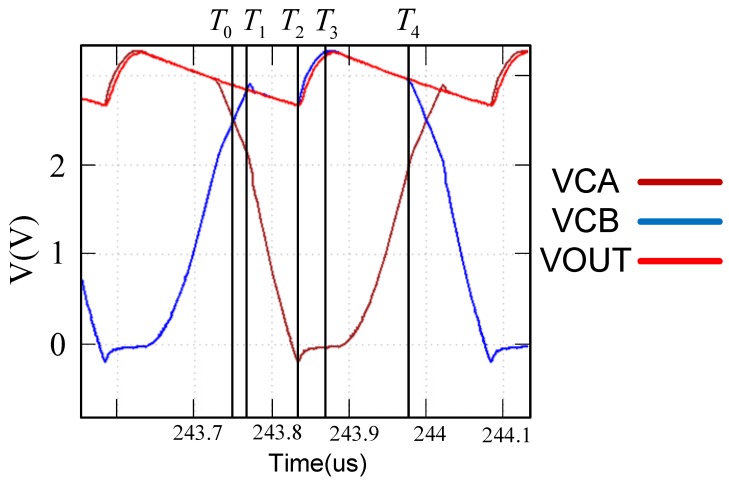
Simulation of voltage vs. time during a cycle.

Figure 12 and Table 1 show the simulation results and conduction switching states of the proposed design. We begin the explanations with T0. VCB and VCA crosses at T0, representing that VCB equals VCA while VCA is higher than GND. Immediately after, VCB becomes higher than VCA in which M1, M4, M6, M8 are OFF while M10 and MP-UP are ON (Table 2), followed by VCB equals VOUT and is higher than VCA, where VCA is still higher than GND (only M1, M10, and MP-UP are ON). VCB and VOUT increase gradually at T2 (VCA < GND), indicating that VOUT is being charged by VCB (only M10 and MP-UP are OFF). At T3, VOUT has been finished charging and starts descending (only M1, M10, and MP-UP are ON), meaning that there is no high-current path as a result of the absence of closed loop. The other cycle begins at T4, where only M10 and MP-UP are ON in order to cut the loop off.

When VCB > VOUT and GND > VCA, M8 is turned on because GND > VCA and the voltage node called a is substantially equal to VCA. VCB > VCA and M1 and M6 are turned on to cause the node called b to approach the voltage of VCB, thus MP-up is in the off state. At this time, the circuit shown in Figure 11 operates on the input signal in the case of charging the load terminal, that is the circuit in Figure 10 turned on in the rectified state. However, when VOUT > VCB and VCA > GND, reverse current is generated thus further damaging circuit performance. However, because M8 and M10 use special process relationships, they will become very sensitive when switching. As long as VCA > GND, it will switch from M8 turn-on/M10 off to M10 turn-on/M8 off. Because M10 is turned on, the voltage of node b will be pulled to GND, and MP-up will be turned off. The voltage at point a is pulled to VOUT, causing the M6 transistor to turn off. However, it is difficult to know whether the transistor of M1 is turned on or off at this time. It is also difficult to determine the size of VCB and VCA. According to the current loop definition, any independent loop in the circuit can define the current flowing through this loop. It can be seen that, when M4 is turned off, the closed loop no longer exists, thus solving the reverse current problem, and M1 should be turned off because neither the closed loop nor the current path exist.

It can be seen from the above analysis that the architecture can prevent reverse current by using a special process for transistor depletion, thus further improving the overall circuit VCE and PCE performance.

**Figure 13:**
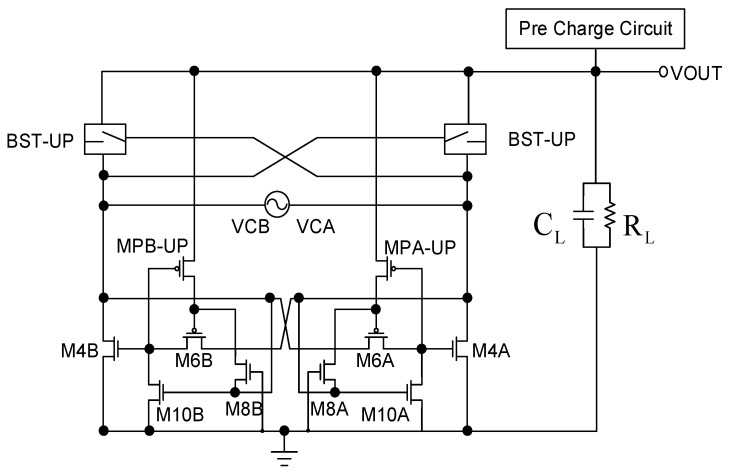
Bootstrap circuit diagram (proposed version).

**Figure 14:**
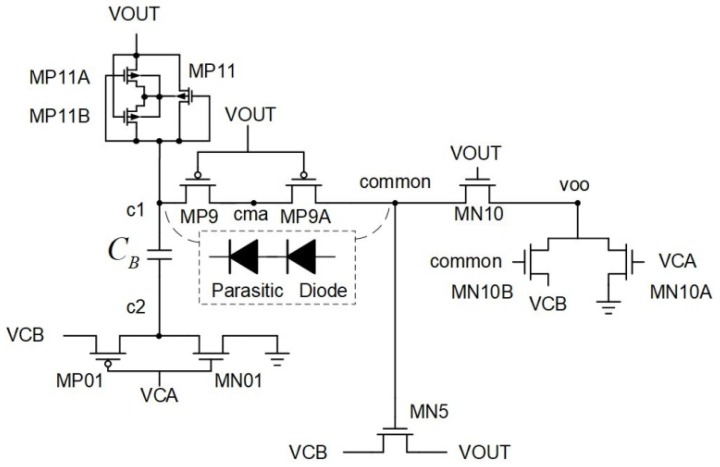
BST-UP (right side).

**Figure 15:**
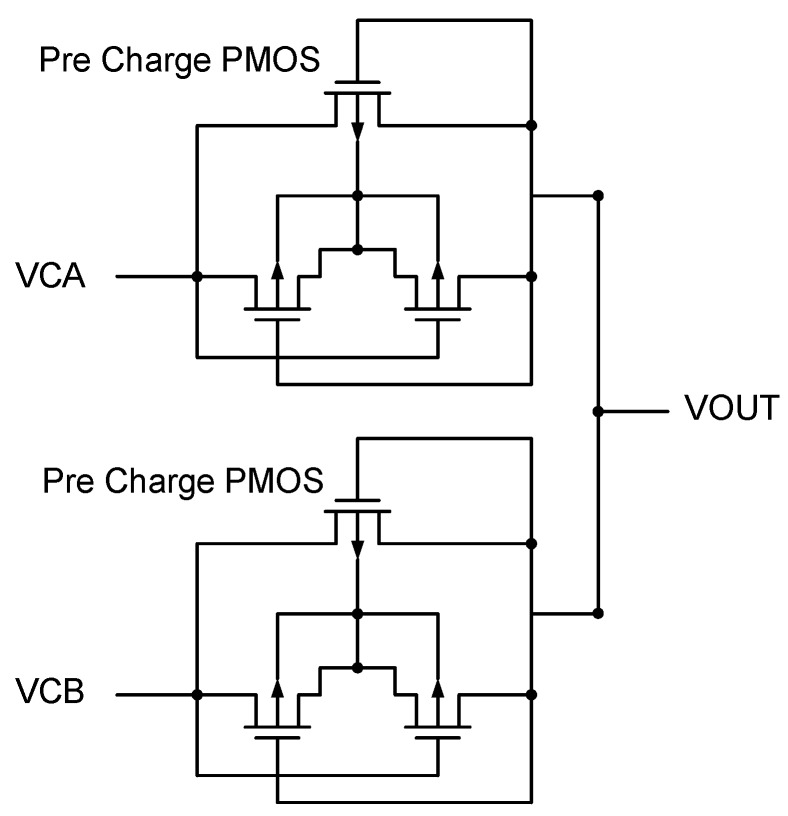
Exterior.

Figure 13, Figure 14 and Figure 15 show the proposed circuit architectures including three major Pre-Charge circuits, BST-UP circuit (Figure 14), and comparator. CL can be charged through the Pre-Charge circuits and Dynamic Bulk Switch. The design concept is based on reference [6] to create an output DC voltage to be used as a power source for the bootstrapped circuit. Then, based on a concept similar to a Voltage Monitor (Figure 9), the DC voltage circuit is converted from originally being supplied by the DC voltage to being supplied by the rectifier output. This not only improves the output DC voltage performance, but also effectively eliminates the breakdown problem. The proposed circuit works as follows:

The Pre-Charge circuits in Figure 15 are constructed with two diode-connected PMOS transistors, resulting in lower conversion efficiency due mainly to the loss of one threshold voltage. This can be solved by means of the BST-UP circuit. When VOUT becomes sufficiently high, the Pre Charge circuits become ineffective in charging VOUT. The BST-UP circuit turns into the one responsible for the task. When VCA > VCB, MP11 and MN01 are ON. The CB can be charged up through VOUT. When VCB > VCA, MP01 is ON so that VCB can be added onto CB in order to conduct MP9 and MP9A, followed by rising the gate of MN5 to be larger than VCB + VTH. By doing so the VCB can only have loss stemming from the inherent resistance of MN5 while charging VOUT. The MP9 an MPA9 have been designed to be series connection in order to decrease the voltage spanned across the MOS terminals. The reverse bias of inherent parasitic diode further enhances the tolerance to overvoltage stress on the MOS. It benefits significantly the reliability of our design as bootstrapped configuration usually comes with penalty of being broken down easily. When VCA re-goes up and becomes higher than VCB, the gate voltage of MN5 will be discharged by the transistors MN10, MN10A, and MN10B. The comparator circuit shown in Figure 13 has the same behavior as that of the circuit in Figure 11, proposed mainly for making the bootstrapped architecture to be capable of having functionality of the active diode. With the depletion type transistors M8A, M8B, M10A, and M10B, comparing behavior can be done through cutting the loop off to avoid reversed current when VOUT is higher than the input voltage, thereby improving the overall VCE and PCE as a whole. 

Here we compare [10] (Figure 11) with the proposed circuit (Figure 13). The architecture resolves the reverse current problem, but the times allowed by the proposed circuit for the conducted transistors of VCA and VCB are clearly smaller than that in [10] (Figure 11). This is mainly due to the bootstrapped architecture of the proposed circuit. A VCB + VCB voltage value is required to eliminate the VTH of MN5 (Figure 14). However, it takes time for VCB to rise, so it is naturally impossible to use cross-coupled PMOS in the architecture proposed in [10] (Figure 11). Section 2.1 shows that the rectifier in the PN architecture better allows the use of a parasitic transistor with a PN junction, making it more likely than the all-N architecture to generate the latch up problem inside the IC. Another benefit of the all-N architecture is its high carrier mobility. Thus, assuming identical conduction currents, the upper half of the N-type architecture will have a smaller crystal than the P-type architecture. This smaller area can be used on POWER MOS to achieve performance close to that shown in Figure 11.

## 3. System Simulation

The resistive load RL of the active circuit system connected to the rear end of the rectifier is determined based on previous findings to determine the equivalent value.

**Figure 16:**
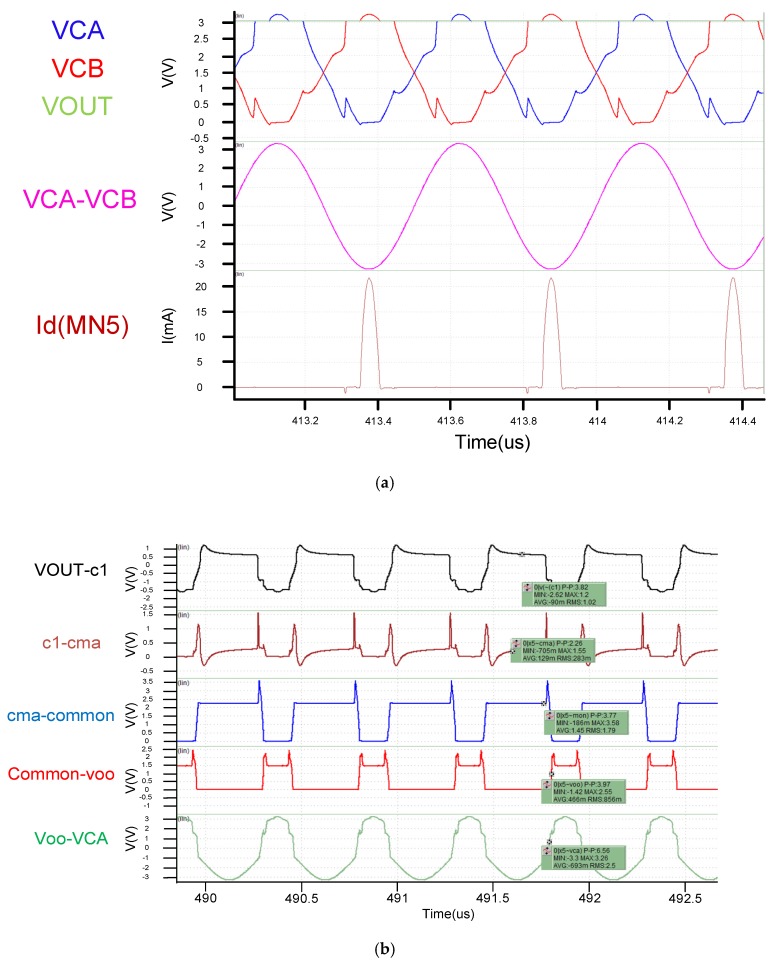
(**a**) Transient simulaiton of proposed rectifier; (**b**) Transient simulation for breakdown check.

The rectifier design specifications are set based on the selected carrier. In this design, RL is set to 1 K Ohm for the assumed average equivalent system load, the carrier frequency is selected to meet the 2 MHz specification, and the load capacitance CL is 1 uF for receiving. The absolute value of the peak voltage across the coil (VCA and VCB) is set to 3.3 V (Figure 16a) for the proposed rectifier following the layout of the above mentioned operating conditions. From Figure 16b knows whether the cross voltage of the each mos (VGS, VGD, VDS) which is drived by boost voltage will breakdown or not in the transient simulation in Figure 14. The cross voltage of the each mos doesn’t over the breakdown voltage (3.6 V) from the waveview. The result is that the transistor elements in the rectifier are respectively operated under the condition that the combination condition of the NMOS and the PMOS is Typical-Typical TT refers to the average error of the carrier mobility of NMOS and PMOS, and represents the average value of the entire MOS drive current. The analog software used is Synopsys’ HSPICE (J-2014.09-2) (Synopsys Inc, Mountain View, CA, USA), while the analog waveform is Synopsys’ Custom WaveView (G-2012.06-SP1) (Synopsys Inc, Mountain View, CA, USA).

**Figure 17:**
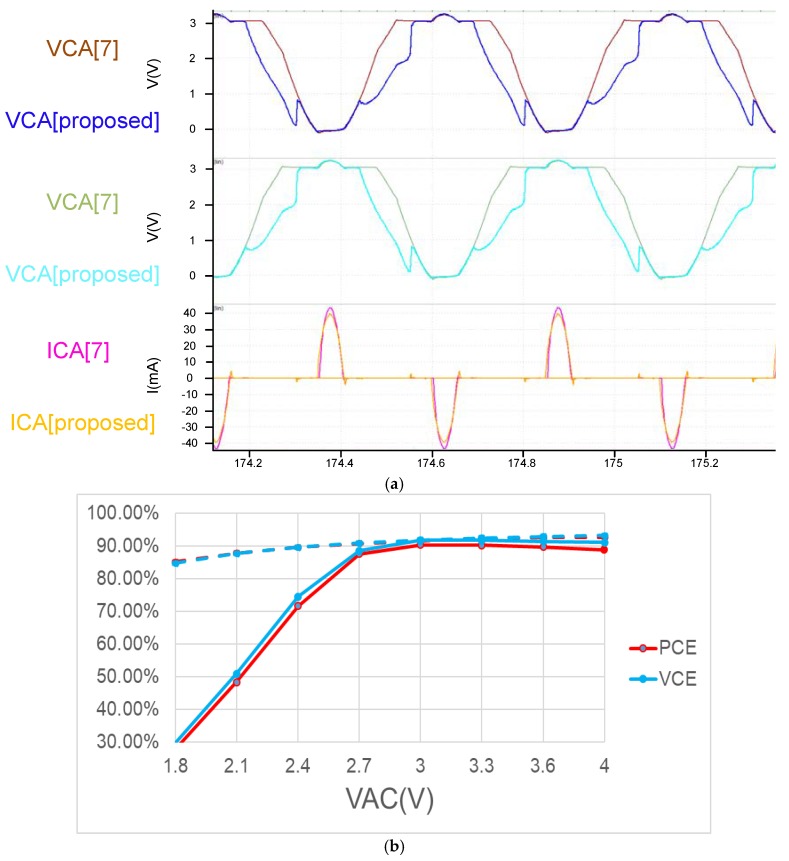
(**a**) Simulation result of comparing [proposed] and [10] (**b**) Compare [proposed] and [7] PCE VCE (solid is proposed circuit dot is [10]) [7] PMOS size is twice that of the upper half of NMOS, operating at 2 MHz with $R_{L}=500\;\Omega$.

From Figure 17a, we see that both the architectures use the same active diode. The architecture is fast, and this result is also reflected in the ICA current in Figure 17a. Also worth noting in Figure 17b is that the two architectures have very different VCE and PCE performances at voltages below 2.7 V. This is mainly because the previously mentioned VCB + VCB voltage of the proposed circuit is sufficiently high to fully utilize circuit performance. However, the proposed circuit offers certain advantages. With Figure 17a,b showing VAC voltages exceeding 2.7 V.

**Figure 18:**
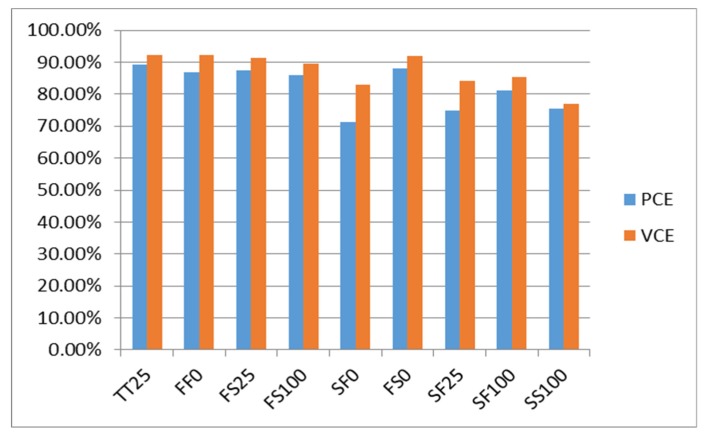
Rectifier conversion efficiency under different process corners.

Figure 18 shows the simulation results of the proposed VCE and PCE on the rectifier under different process corners. To objectively and correctly evaluate the performance of the proposed rectifier, we define the voltage conversion efficiency according to the component performance evaluation standard depending on the general electronic design:

(1) $$VCE=\frac{VOUT}{VIN}\cdot 100\%$$

(2) $$\begin{array}{c} \mathrm{PCE}=\frac{P_{\mathrm{OUT}}}{P_{\mathrm{OUT}}+P_{\mathrm{DISS}}}\cdot 100\%\\ =\frac{I_{RL}{}^{2}\cdot R_{L}}{I_{RS}{}^{2}\cdot R_{S}\cdot D+I_{RL}{}^{2}\cdot R_{L}}\cdot 100\% \end{array}$$

**Figure 19:**
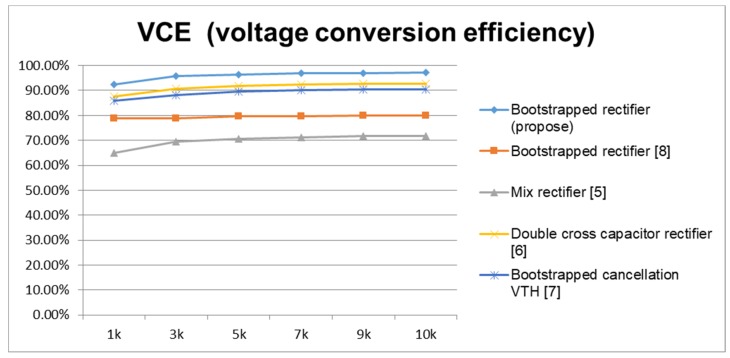
Adjusting the VCE RL for various rectifiers.

**Figure 20:**
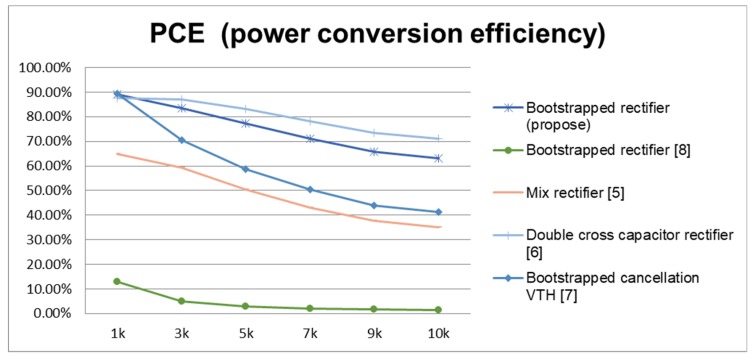
Adjusting the PCE RL for various rectifiers.

From Figure 19 and Figure 20, we define the conversion efficiency by objectively comparing a previous case with the same specifications, and normalizing the size of the main rectifier power transistor. From the simulation analysis, it can be seen that, regardless of the process offset, the rectifier design changes under the condition that the resistive load of the active circuit is later changed. Both have relatively high and stable conversion performance. Similarly, we adjust the carrier frequency to observe the changes in conversion performance for each architecture. The upper limit of the carrier frequency is the equivalent resistance and capacitance when the transistor size is adjusted in the architecture. Care must be taken to meet the design requirements, such that the need to select the carrier frequency can be taken into account before the conversion performance can be maximized.

## 4. Measurement and Wafer Implementation

**Figure 21:**
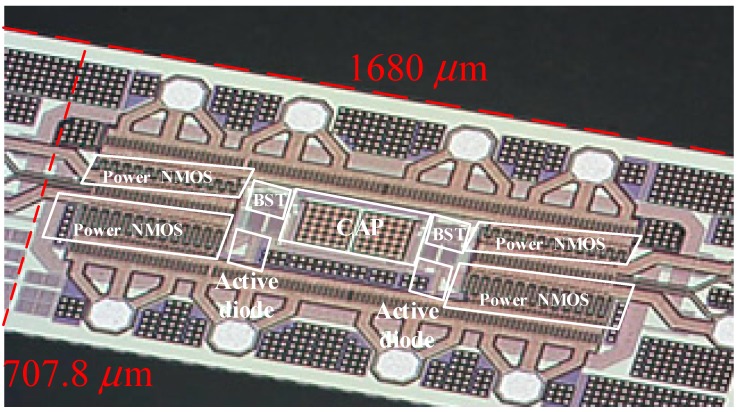
Chip die photo.

This section introduces the proposed circuit measurement setup. The input signal uses a Signal Generator (Agilent 33522B Waveform Generator) (Agilent Technologies, Santa Clara, CA, USA) to provide a set of 3.3 V AC frequency sine wave signal with a 2 MHz AC sinusoidal signal connected at one end of the coil and the other end to the VCA and VCB. In the above, the signal generated by the Signal Generator causes the coupled coil to have mutual inductance, so the signal is transmitted from one end of the coupled coil to the other end to the input contacts of the VCA and VCB. During measurement, the output line from the chip to the connector end on the PCB should be the same length and width, and the signal line used to connect to the oscilloscope (MSO7014A Mixed Signal Oscilloscope (Keysight, Santa Rosa, CA, USA)) must be the same length. Otherwise, it will result in different attenuation, which will cause amplitude error. However, due to insufficient output power of the signal generator itself, the chip itself does not have sufficient input power, resulting in a large extraction at the output. As a result, we require a power amplifier to provide the chip sufficient power for reaching the target output voltage. The proposed active rectifier was designed and fabricated in 180-nm (3.3 V device) CMOS process. The chip die photo of the active rectifier is shown in Figure 21. The size of this chip, including pads and seal ring, is 1.189 mm^2^, and the active rectifier area is 0.3996 mm^2^.

### 4.1. Power Amplifier

**Figure 22:**
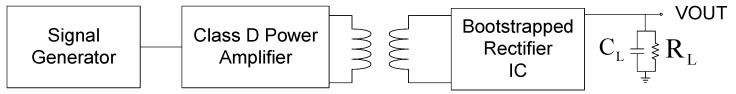
Test system block diagram.

**Figure 23:**
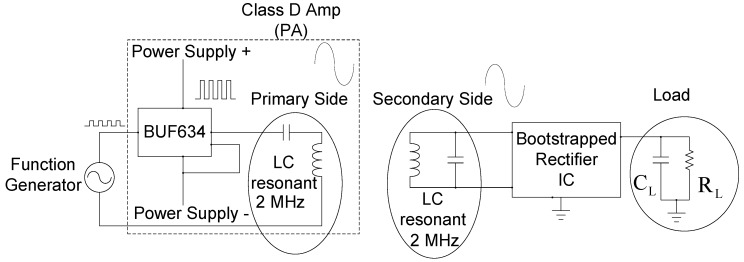
Measurement setup.

A power amplifier mainly amplifies the output waveform energy of the signal generator, and then transfers the signal from the primary side to the secondary input wafer by means of the charge coil. The schematic diagram is shown in Figure 22 and Figure 23.

Figure 23 mainly uses the Function Generator and the IC of BUF634, which is mainly a buffer circuit that can amplify the signal power of the Function Generator through the Power Supply. Because of needing the 2 MHz sinusoidal at the input signal, we need LC resonant to generate 2 MHz frequency band. Finally, the energy is transmitted through the resonant charge of the primary side and the secondary side, the output mode energy of the signal generator can be amplified.

**Figure 24:**
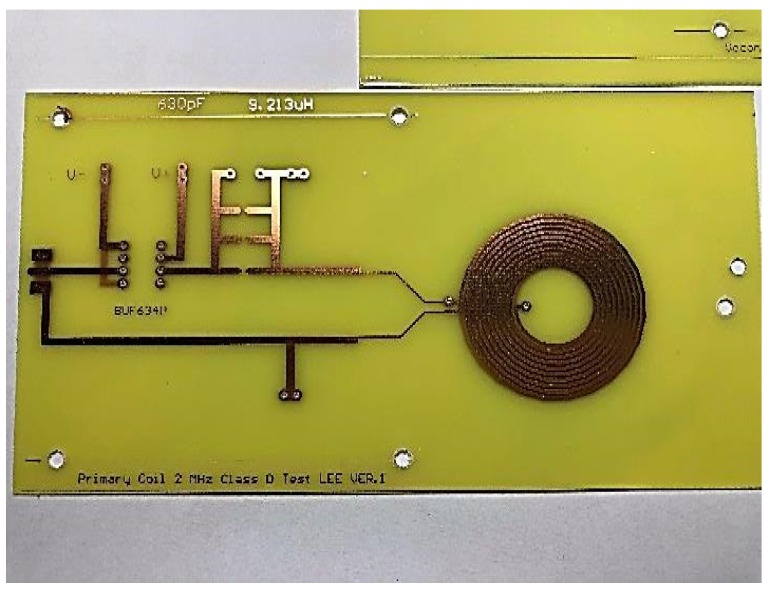
Primary measurement resonance coil.

**Figure 25:**
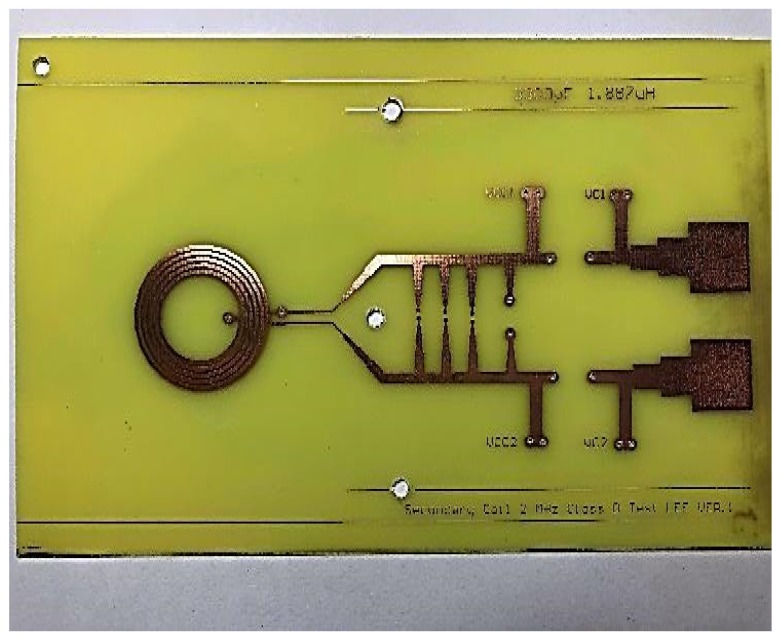
Secondary measurement resonance coil.

**Figure 26:**
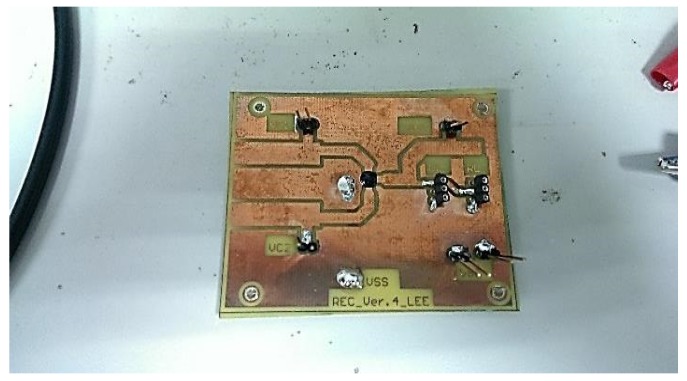
Bootstrapped Rectifier IC and its PCB.

Figure 24 and Figure 25 show the primary and secondary coils in printed circuit board. Their specifications along with the devices forming the resonant network are tabulated in Table 2. Figure 26 shows the implemented circuit board used for the measurement of proposed bootstrapped rectifier chip.

### 4.2. Measurement Results

**Figure 27:**
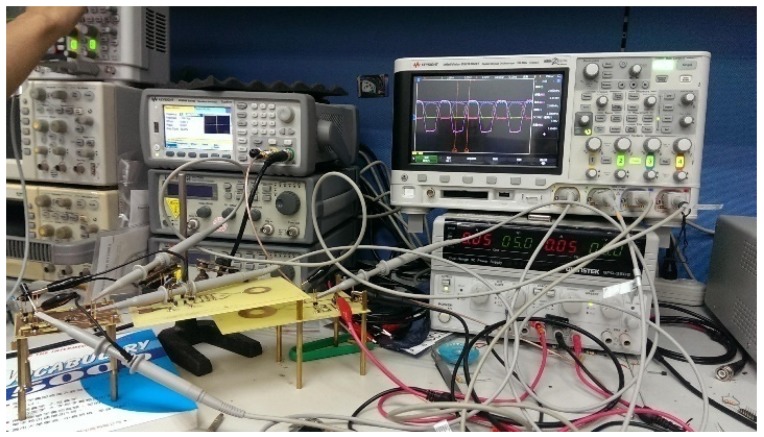
Actual measurement board (overall system).

**Figure 28:**
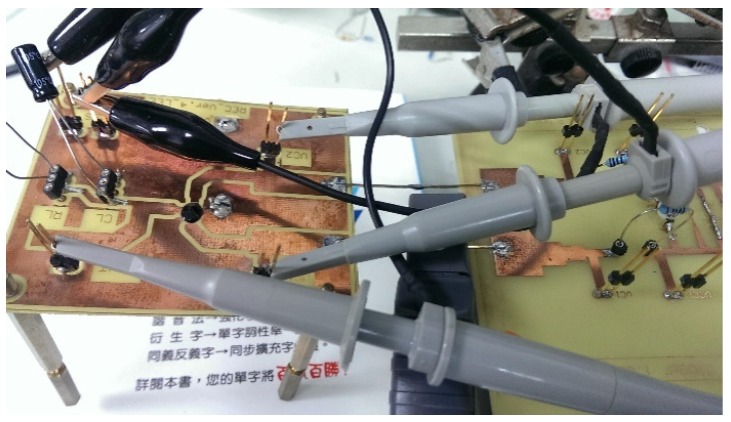
Actual measurement board (IC).

**Figure 29:**
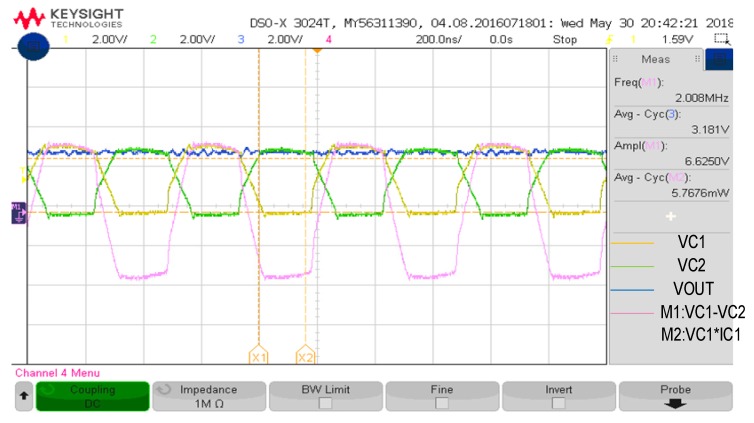
Actual measurement board ($R_{L}=1\;k\Omega$).

**Figure 30:**
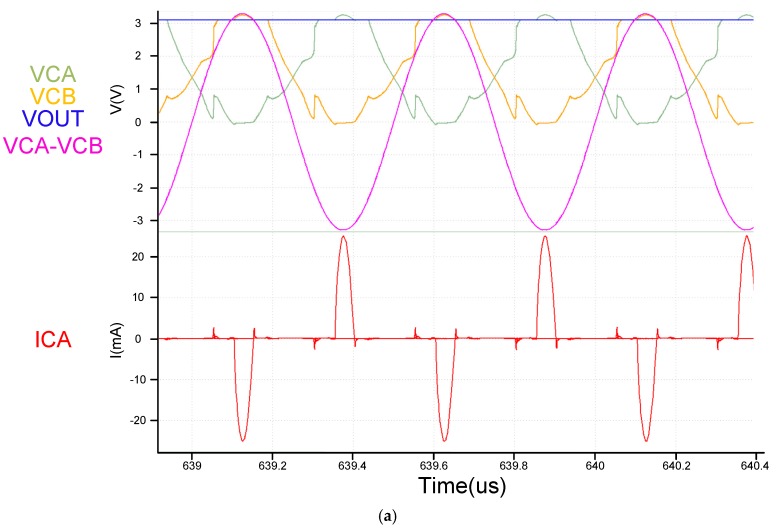

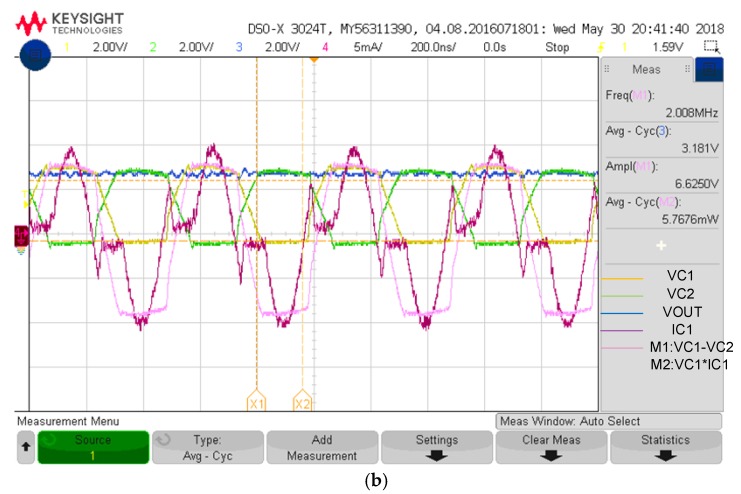
(**a**) simulation waveforms ($R_{L}=1\;k\Omega$ including input current); (**b**) Actual measurement board ($R_{L}=1\;k\Omega$ including input current).

**Figure 31:**
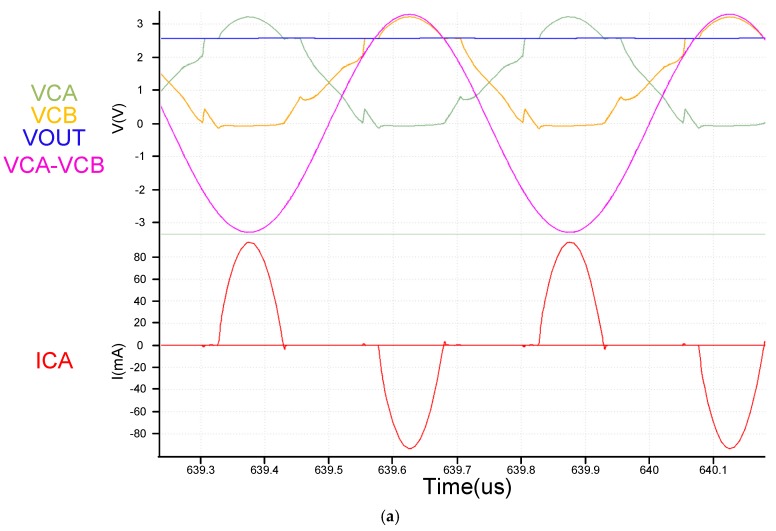

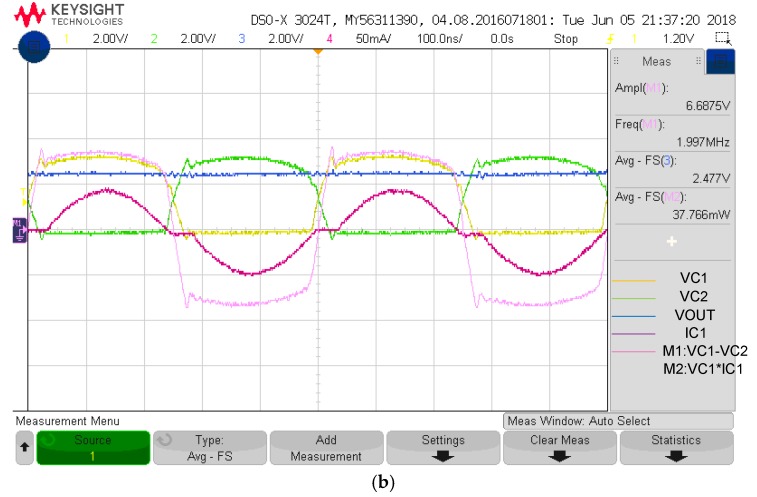
(**a**) simulation waveforms ($R_{L}=100\;\Omega$ including input current); (**b**) Actual measurement board ($R_{L}=100\;\Omega$ including input current).

**Figure 32:**
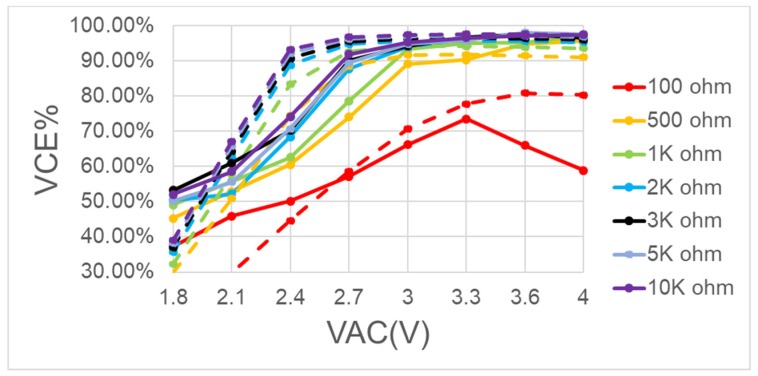
Measured voltage conversion efficiency of the BST rectifier with different loadings (solid is measured, dot is simulation).

After the above mentioned system is completed, the actual board measurement results are shown in Figure 27 and Figure 28. Figure 29 and Figure 30 are the measurement waveforms of the fabricated chip. The measurement is under loading combination of $R_{L}=1\;k\Omega ,C_{L}=1\;\mathrm{uF}$, with 3.3-V sinusoidal VC1 and VC2 (VC1 = VCA, VC2 = VCB, VCA and VCB from Figure 13) of 2 MHz (loading current 3.181 mA). From the waveforms, it can be seen that VC1(yellow) and VC2(green) behave like perfect rectified waveforms, where VOUT (blue) has ripple-less voltage thanks to the low-pass filtering. Figure 31 are the measured waveforms under the same conditions excluding the loading combination, where the combination was set as $R_{L}=100\;\Omega ,C_{L}=1\;\mathrm{uF}$. By comparing the results of Figure 30 and Figure 31, it can be seen that the current (crimson) waveforms of them are a little bit different. In Figure 30, switching delays occur in the transistors M4 and M10 of Figure 13 at approximately the cross point of VC1 and VC2, causing reverse current, due to light load (the active diodes were not turned on in time). In Figure 31, however, the overall response becomes faster due to the heavy load. This makes the measurement matches the simulation and benefits the switching of M4 and M10 in Figure 13 for reducing the delay. Moreover, the heavy load setup alleviates the interference caused by the noise in measurement, thereby smoothing the measured waveforms in display. Both PCE and VCE are critical performance indexes. In Figure 32, it can be seen that the circuit has its best VCE at near 3.3 V under different input voltages and loads.

From the figure, we see that VCE worsens as the load grows more severe, mainly because, given a power transistor with a fixed size and a fixed driving voltage, a larger current load will produce a higher internal resistance pressure drop. Figure 32 shows that, the lighter the load, the better the VCE conversion efficiency.

**Figure 33:**
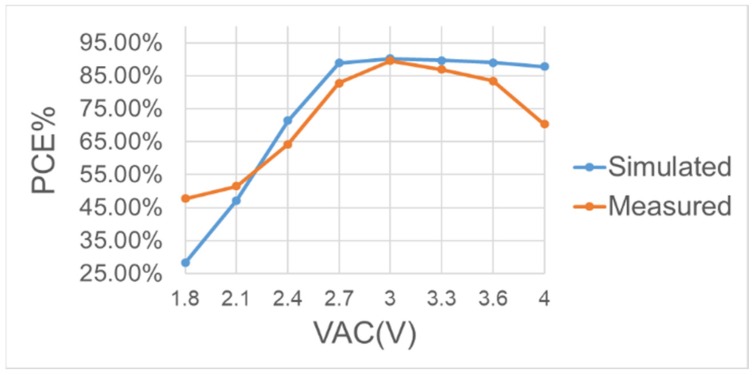
Measured and simulated power conversion efficiency of the BST rectifier operating at 2 MHz with $R_{L}=1\;k\Omega$.

**Figure 34:**
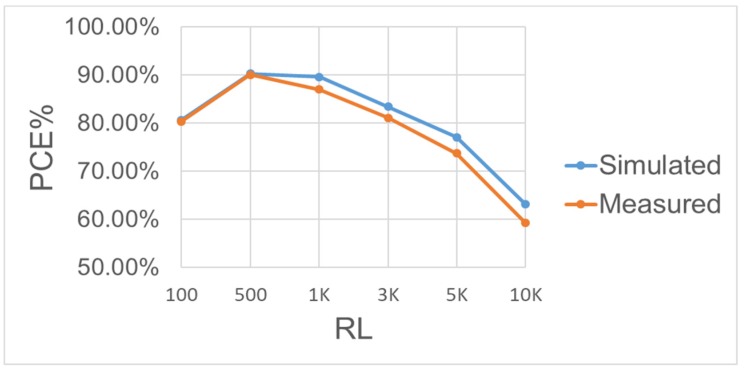
Measured and simulated power conversion efficiency of the BST rectifier operating at 2 MHz with VAC = |3.3 V| and different loadings.

**Figure 35:**
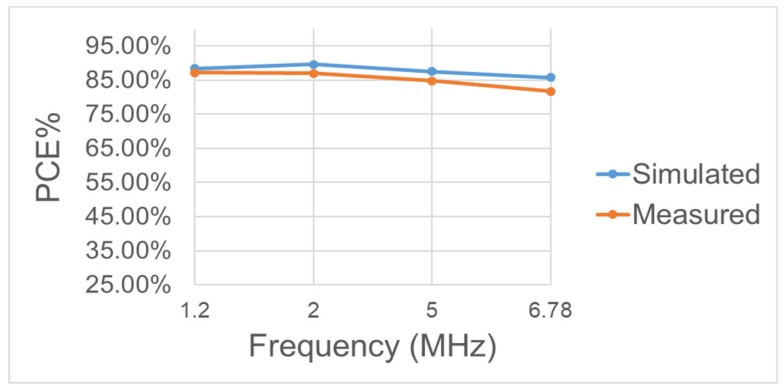
Measured and simulated power conversion efficiency of the BST rectifier operating at different frequencies with VAC = |3.3 V|, $R_{L}=1\;k\Omega$.

Figure 33, Figure 34 and Figure 35 show different PCE under different conditions for the comparison of the simulation and measurement. Figure 33 shows how the input VAC affects PCE. From the curves, it can be seen that the bootstrapped cannot be fully functional when the input is below 2.7 V, causing that the M5 of Figure 14 does not have sufficient high voltage to turn on, and consequently affect the output. For every input higher than 3.3 V, there will be a best geometry ratio to achieve best efficiency. There will be loss once the input is far beyond or lower than the preset input, due to switching power dissipation caused by the parasitic effect.

Figure 34 shows the PCE under different $R_{L}$(100 Ω–10 kΩ). It can be seen that the PCE decreases with $R_{L}$. The dominant reason can be attributed less consumed current in the bootstrapped circuit and main conducting transistors. The architecture in Figure 14 shows that the Power MOS in the rectifier circuit mainly operates in a linear region and is used as a voltage input/output switch. However, as a switch, the transistor’s internal resistance is a concern. The key factors in determining resistance are the size of the two crystals and the VGS voltage difference. We first discuss the VGS. The proposed circuit mainly boosts the power transistor using the CB storage voltage. Therefore, the size of the CB capacitor will affect the size of the VGS and the switches’ internal resistance and thus the crystal size. The larger the transistor, the smaller the internal resistance, but the larger the parasitic cap. Thus, the size of the CB capacitor must be considered to meet the application design size and CB capacity, thus the best PCE locates between 100 Ω and 3 kΩ for $R_{L}$. From Figure 35, one can learn that PCE is influenced by the frequency. With the same working conditions, the proposed rectifier has its best PCE at 2 MHz. For different frequencies, the equivalent resistance and parasitic capacitance of main transistors will vary and the switching loss depending on them will be different. Another major effect associated with the varied resistance and capacitance is the occurrence of reverse current. The design has been optimized in terms of its performance for a 2-MHz frequency in spite of its capability of achieving very good conversion efficiency for a wide range of operation frequencies. Table 3 compares the proposed design with other state-of-the-art works. It can be obvious that this work has higher PCE and VCE.

Table 3 summarizes the three parameters of VCE, PCE and Core Area, showing that when VCE is poor, PCE will be worse. This is mainly because the poorer VCE means a larger voltage across the power transistor, indicating greater energy consumption by the power transistor. Therefore, PCE will worsen with the poor VCE reference because the power transistor consumes a smaller crossover voltage, but the active diode switching is not sufficiently accurate. In this case, greater reverse current will result in extra unnecessary power consumption, further worsening the PCE conditions. Finally, the last part discusses the Core Area. As previously mentioned, better VCE requires smaller cross-voltage, but this is related to the structural design and the crystal size. The larger the transistor, the smaller the internal resistance, and the larger the POUT, MAX. Thus, for the rectifier circuit, the theoretically larger Core Area will improve performance. The conversion efficiency will vary with the circuit structure, operating frequency, and output loading. To achieve optimal efficiency, there are generally two points to be considered in designing a rectifier circuit. The first is the geometry ratio of the power MOSFET. With proper sizing, the driving capability can be maximized. Tradeoffs have to be made between the parasitic capacitance and conductance. Secondly a rectifier circuit has to control accurately the turn on/off timing to avoid the reverse current in order to reduce power/energy consumption.

Presented is a novel all-N rectifier circuit for energy harvesting applications. The design features low conduction resistance due mainly to the all N-type MOSFETs as the power transistors. The all-N configuration also substantially reduces the latch-up, while at the same time decreasing the implementation silicon area. With the dynamic bulk modulation and the body biasing, the breakdown concern stemming from the step up of the bootstrapped circuit structure can be dramatically avoided. The results from the actual chip tests reveal that its efficiency can be quite competitive with those of p-type transistors currently demonstrated in the literature.

## 5. Conclusions

We have presented in this paper a novel bootstrapped rectifier with N-type power transistors with active diodes for implantable systems. A pre-charge mechanism based on bulk modulation has been used to prevent latch up and decrease leakage current. With the advantages of the bootstrapped technique, the power transistors can be fully conductive to reduce the loss of its internally equivalent resistance, thereby maximizing the conversion efficiency. The enhanced-mode transistors will help the power transistors behave as active diode when the output voltage is higher than the input voltage, thereby dramatically decreasing the reverse current. The conversion efficiency can be improved by means of the power transistors functioning normally when the input voltage is high than the output voltage. This novel all-N design features several advantages. Implemented in the TSMC standard 0.18-μm 3.3-V CMOS, the proposed deign has been proven to a quite competitive solution as compared with those state-of-the-art works demonstrated in the literature.

## Figures and Tables

**Table 1:** Rectifier Switching State during Cycle.

	M1	M4	M6	M8	M10	M_P-UP_
T0	OFF	OFF	OFF	OFF	ON	ON
T1	ON	OFF	OFF	OFF	ON	ON
T2	ON	ON	ON	ON	OFF	OFF
T3	ON	OFF	OFF	OFF	ON	ON
T4	OFF	OFF	OFF	OFF	ON	ON

T = time, M = MOS Transistor (gold-oxygen half field effect transistor).

**Table 2:** PCB Coil Parameter Data.

	Primary	Secondary
Total inductance—Circular	9.213 μH	1.887 μH
LC sensor capacitance	620 pF	3300 pF
Outer diameter of inductor	37.5 mm	26.5 mm
Coil inner diameter	15 mm	15 mm
Turns per layer	10 turns	5 turns
Trace width	0.8 mm	0.8 mm
Spacing between traces	0.3 mm	0.3 mm
Q Factor	83.253	41.921
Resonance impedance	10,250.177 Ω	1003.75 Ω
AC/DC resistance	1.45 Ω/1.009 Ω	0.57 Ω/0.399 Ω

**Table:** (PART A)

Reference	[9]	[10]	[11]	[12]	[7]	[13]	[14]
**Year**	2008	2009	2009	2011	2012	2012	2014
**Technology**	0.5 μm (CMOS)	0.18 μm (CMOS)	0.35 μm (CMOS)	0.5 μm (CMOS)	0.18 μm (CMOS)	0.18 μm (CMOS)	0.18 μm (CMOS)
**Input Voltage**	5 V	2.5 V (Peak to Peak)	1.2 V–2.4 V	3.8 V	0.8 V; 1.8 V; 2.7 V	1.5	1.192 V
**Frequency**	0.1–2 MHz	1 MHz	200 kHz–1.5 MHz	13.56 MHz	10 MHz	13.56 MHz	13.56 MHz
**Maximum Output Voltage**	4.36 V	NA	1.13 V–2.28 V (R_L_ = 2 kΩ) 0.98 V–2.08 V (R_L_ = 100 Ω)	3.12 V	0.5 V, 0.3 V * 1.5 V, 1.2 V * 2.3 V, 2 V *	1.33	0.808 V
**VCE**	87.2%	NA	95%	82.11%	62.5%, 37.5% * 83%, 66.7% * 85.18%, 74.07% *	88.67%	67.79%
**P_OUT,MAX_**	NA	NA	NA	NA	2 mW	NA	40 mW
**Core Area**	2.25 mm^2^	1.72 mm^2^	1.03 mm^2^	0.18 mm^2^	0.608 mm^2^	0.009 mm^2^	0.12 mm^2^ (Without PAD)
**PCE**	84.8% (0.5 MHz)	76%	82–87% (R_L_ = 100 Ω)	81.9%	69%, 37% * 83%, 71% * 86%, 80% *	80.2%	85% (@R_L_ = 100 Ω)

**Table:** (PART B)

Reference	[15]	[16]	[17]	[18]	[19]	This Work
**Year**	2014	2015	2016	2016	2016	2018
**Technology**	0.35 μm (CMOS)	0.35 μm (CMOS)	0.35 μm (CMOS)	0.65 μm	0.35 μm	0.18 μm (CMOS)
**Input Voltage**	1.5 V–4 V	NA	1.8 V–3.6 V	1.3 V–2.5 V	N/A	2.7 V–4 V
**Frequency**	13.56 MHz	13.56 MHz	13.56 MHz	13.56 MHz	6.78 MHz	2 MHz
**Maximum Output Voltage**	1.28 V–3.56 V (R_L_ = 1.8 kΩ) 1.19 V–3.52 V (R_L_ = 500 Ω)	3.6 V	3.405 V	1.24 V–2.44 V	5 V	2.123 V *–3.888 V * (@R_L_ = 1 kΩ)
**VCE**	89%	NA	91.2–94.6% (@R_L_ = 100–1 kΩ) 90.4–92.4% (@R_L_ = 500 Ω)	94.8–97.7% (@R_L_ = 500 Ω) 91.7–95.2% (@R_L_ = 100 Ω)	N/A	73.55 *–95.12% * (@R_L_ = 100–1 kΩ) (@VAC = 3.3 V)
**P_OUT,MAX_**	24.8 mW	102 mW	64.8 mW	248.1 mW	6 W	61.35 mW
**Core Area**	0.186 mm^2^	3.06 mm^2^	1.134 mm^2^	1.44 mm^2#^	4.77 mm^2^	1.189 mm^2^ (with pad and seal ring)
**PCE**	82.2–90.1% (R_L_ = 500 Ω)	92.6%	89.1–91.4% (@R_L_ = 500 Ω)	88.5~91% (@R_L_ = 500 Ω) 91.3–94.6% (@R_L_ = 100 Ω)	92.2% (@P_OUT_ = 3.5 W)	80.36 *–90.08% * (@R_L_ = 100–1 kΩ) (@VAC = 3.3 V)

Comparison of previously referenced rectifiers where * indicates measurement results comparison of previously referenced rectifiers where ^#^ indicates Integrated output capacitor C_O_ (4.8 nF) include.
